# Polymorphisms in transcription factor binding sites and enhancer regions and pancreatic ductal adenocarcinoma risk

**DOI:** 10.1186/s40246-024-00576-x

**Published:** 2024-02-02

**Authors:** Pelin Ünal, Ye Lu, Bas Bueno-de-Mesquita, Casper H. J. van Eijck, Renata Talar-Wojnarowska, Andrea Szentesi, Maria Gazouli, Edita Kreivenaite, Francesca Tavano, Ewa Małecka-Wojciesko, Bálint Erőss, Martin Oliverius, Stefania Bunduc, Mateus Nóbrega Aoki, Ludmila Vodickova, Ugo Boggi, Matteo Giaccherini, Jurate Kondrackiene, Roger Chammas, Orazio Palmieri, George E. Theodoropoulos, Maarten F. Bijlsma, Daniela Basso, Beatrice Mohelnikova-Duchonova, Pavel Soucek, Jakob R. Izbicki, Vytautas Kiudelis, Giuseppe Vanella, Paolo Giorgio Arcidiacono, Barbara Włodarczyk, Thilo Hackert, Ben Schöttker, Faik G. Uzunoglu, Franco Bambi, Mara Goetz, Viktor Hlavac, Hermann Brenner, Francesco Perri, Silvia Carrara, Stefano Landi, Péter Hegyi, Frederike Dijk, Evaristo Maiello, Giovanni Capretti, Sabrina Gloria Giulia Testoni, Maria Chiara Petrone, Hannah Stocker, Stefano Ermini, Livia Archibugi, Manuel Gentiluomo, Giulia Martina Cavestro, Raffaele Pezzilli, Gregorio Di Franco, Anna Caterina Milanetto, Cosimo Sperti, John P. Neoptolemos, Luca Morelli, Klara Vokacova, Claudio Pasquali, Rita T. Lawlor, Francesca Bazzocchi, Juozas Kupcinskas, Gabriele Capurso, Daniele Campa, Federico Canzian

**Affiliations:** 1 Genomic Epidemiology Group, German Cancer Research Center, In Neuenheimer Feld 280, 69120 Heidelberg, Germany; 2 Department for Determinants of Chronic Diseases, National Institute for Public Health and the Environment, Bilthoven, The Netherlands; 3 Department of Surgery, Erasmus MC University Medical Center, Rotterdam, The Netherlands; 4 Department of Digestive Tract Diseases, Medical University of Lodz, Lodz, Poland; 5 Institute for Translational Medicine, Medical School, University of Pécs, Pécs, Hungary; 6 János Szentágothai Research Center, University of Pécs, Pécs, Hungary; 7 Laboratory of Biology, Medical School, National and Kapodistrian University of Athens, Athens, Greece; 8 Gastroenterology Department and Institute for Digestive Research, Lithuanian University of Health Sciences, Kaunas, Lithuania; 9 Division of Gastroenterology and Research Laboratory, Fondazione IRCCS “Casa Sollievo della Sofferenza” Hospital, San Giovanni Rotondo, FG Italy; 10 Center for Translational Medicine, Semmelweis University, Budapest, Hungary; 11 Division of Pancreatic Diseases, Heart and Vascular Center, Semmelweis University, Budapest, Hungary; 12 Department of Surgery, University Hospital Kralovske Vinohrady, Third Faculty of Medicine, Charles University, Prague, Czech Republic; 13 Carol Davila University of Medicine and Pharmacy, Bucharest, Romania; 14 Laboratory for Applied Science and Technology in Health, Carlos Chagas Institute, Curitiba, PR Brazil; 15 Biomedical Center, Faculty of Medicine in Pilsen, Charles University, Plzeň, Czech Republic; 16 Department of Molecular Biology of Cancer, Institute of Experimental Medicine of the Czech Academy of Sciences, Prague, Czech Republic; 17 Institute of Biology and Medical Genetics, Institute of Physiology, 1st Faculty of Medicine, Charles University, Prague, Czech Republic; 18 Division of General and Transplant Surgery, Pisa University Hospital, Pisa, Italy; 19 Department of Biology, University of Pisa, Pisa, Italy; 20 Department of Radiology and Oncology, Institute of Cancer of São Paulo, São Paulo, Brazil; 21 First Propaedeutic University Surgery Clinic, Hippocratio General Hospital, Medical School, National and Kapodistrian University of Athens, Athens, Greece; 22 Laboratory for Experimental Oncology and Radiobiology, Center of Experimental Molecular Medicine, Amsterdam UMC Location University of Amsterdam, Amsterdam, the Netherlands; 23 Cancer Center Amsterdam, Imaging and Biomarkers, Amsterdam, the Netherlands; 24 Department of Medicine, Laboratory Medicine, University of Padova, Padua, Italy; 25 Department of Oncology, Faculty of Medicine and Dentistry, Palacky University, Olomouc, Czech Republic; 26 Department of General Visceral and Thoracic Surgery, University of Hamburg Medical Institutions, Hamburg, Germany; 27 PancreatoBiliary Endoscopy and Endosonography Division, Pancreas Translational and Clinical Research Center, San Raffaele Scientific Institute, Milan, Italy; 28 Digestive and Liver Disease Unit, S. Andrea Hospital, Rome, Italy; 29 Department of General, Visceral and Transplant Surgery, Heidelberg University Hospital, Heidelberg, Germany; 30 Division of Clinical Epidemiology and Aging Research, German Cancer Research Center, Heidelberg, Germany; 31 Network Aging Research (NAR), Heidelberg University, Heidelberg, Germany; 32 Blood Transfusion Service, Meyer Children’s Hospital, Florence, Italy; 33 Division of Preventive Oncology, German Cancer Research Center and National Center for Tumor Diseases, Heidelberg, Germany; 34 German Cancer Consortium (DKTK), German Cancer Research Center, Heidelberg, Germany; 35 Endoscopic Unit, Department of Gastroenterology, IRCCS Humanitas Research Hospital, Milan, Italy; 36 Department of Pathology, Cancer Center Amsterdam, Amsterdam UMC, University of Amsterdam, Amsterdam, the Netherlands; 37 Department of Oncology, Fondazione IRCCS “Casa Sollievo della Sofferenza” Hospital, San Giovanni Rotondo, FG Italy; 38 Pancreatic Unit, IRCCS Humanitas Research Hospital, Milan, Italy; 39 Department of Biomedical Sciences, Humanitas University, Milan, Italy; 40 Gastroenterology and Gastrointestinal Endoscopy Unit, IRCCS San Raffaele Scientific Institute, Vita-Salute San Raffaele University, Milan, Italy; 41 Department of Gastroenterology, San Carlo Hospital, Potenza, Italy; 42 General Surgery Unit, Department of Translational Research and New Technologies in Medicine and Surgery, University of Pisa, Pisa, Italy; 43 Department of Surgery, Oncology and Gastroenterology, University of Padova, Padua, Italy; 44 Department of Diagnostics and Public Health, ARC-Net Centre for Applied Research on Cancer, University of Verona, Verona, Italy; 45 Department of Surgery, Fondazione IRCCS “Casa Sollievo della Sofferenza” Hospital, San Giovanni Rotondo, FG Italy

**Keywords:** Association study, Enhancer, Pancreatic cancer, Single nucleotide polymorphism, Transcription factor binding site

## Abstract

Genome-wide association studies (GWAS) are a powerful tool for detecting variants associated with complex traits and can help risk stratification and prevention strategies against pancreatic ductal adenocarcinoma (PDAC). However, the strict significance threshold commonly used makes it likely that many true risk loci are missed. Functional annotation of GWAS polymorphisms is a proven strategy to identify additional risk loci. We aimed to investigate single-nucleotide polymorphisms (SNP) in regulatory regions [transcription factor binding sites (TFBSs) and enhancers] that could change the expression profile of multiple genes they act upon and thereby modify PDAC risk. We analyzed a total of 12,636 PDAC cases and 43,443 controls from PanScan/PanC4 and the East Asian GWAS (discovery populations), and the PANDoRA consortium (replication population). We identified four associations that reached study-wide statistical significance in the overall meta-analysis: rs2472632(A) (enhancer variant, OR 1.10, 95%CI 1.06,1.13, *p* = 5.5 × 10^−8^), rs17358295(G) (enhancer variant, OR 1.16, 95%CI 1.10,1.22, *p* = 6.1 × 10^−7^), rs2232079(T) (TFBS variant, OR 0.88, 95%CI 0.83,0.93, *p* = 6.4 × 10^−6^) and rs10025845(A) (TFBS variant, OR 1.88, 95%CI 1.50,1.12, *p* = 1.32 × 10^−5^). The SNP with the most significant association, rs2472632, is located in an enhancer predicted to target the *coiled-coil domain containing 34* oncogene. Our results provide new insights into genetic risk factors for PDAC by a focused analysis of polymorphisms in regulatory regions and demonstrating the usefulness of functional prioritization to identify loci associated with PDAC risk.

**Supplementary Information:**

The online version contains supplementary material available at 10.1186/s40246-024-00576-x.

## Introduction

Despite rapid advances in modern medical technology and significant improvements in survival rates of many cancers, pancreatic ductal adenocarcinoma (PDAC) is still highly lethal, with a 5-year survival after diagnosis of 11% [1]. PDAC is rarely detected at an early stage, and its etiology is still not completely clear [2, 3]. As a consequence, there is an urgent need to construct a successful PDAC risk assessment model to identify susceptible individuals for prevention or early detection and advance our understanding of pancreatic carcinogenesis. The ultimate goal is to reduce the incidence and mortality of PDAC.

Cigarette smoking, increased body mass index, heavy alcohol consumption, and a diagnosis of diabetes mellitus have all been demonstrated to increase the risk of PDAC [4]. Family history of pancreatic cancer has been associated with increased risk, suggesting that inherited genetic factors also play an essential role, with approximately 5–10% of PDAC patients reporting a family history of pancreatic cancers [5].

Among inherited genetic factors, single-nucleotide polymorphisms (SNPs) are the most frequently studied variations, mainly in genome-wide association studies (GWAS). Thanks to GWAS, many associations of genome-wide significance (*p* < 5 × 10^−8^) have been reported between genetic variants and common diseases and traits [6]. These associations have led to insights into the architecture of disease susceptibility which might lead to advances in clinical care and personalized medicine. The number of independent susceptibility variants for PDAC has been estimated to be nearly 2000 according to a method to estimate the degree of polygenicity [7]; however, only 30 independent loci at genome-wide significance level have been discovered so far [8]. Therefore, a large number of PDAC risk SNPs remains to be found [9, 10].

Much research is focused on genetic variants in protein-coding regions because their potential impact on proteins is relatively easy to predict; however, the majority of risk variants are located in non-coding regions. Non-coding variants are unrelated to the final amino acid sequences and protein functions such as DNA binding, catalytic activity, and ligand–receptor interaction. However, the possible effect of these variants is differential gene expression.

Secondary analyses have been conducted on existing GWAS data to identify novel loci, and additional cases and controls have been genotyped from independent populations, which has also been implemented successfully on PDAC. For instance, we and others have successfully investigated the association of SNPs in long noncoding RNAs (lncRNAs) [11], microRNAs [12], expression quantitative trait loci (eQTLs) [13] and particular pathway-related genes [14–16] for PDAC, resulting in several novel germline risk loci.

Here, we focused on genetic variants located in two major types of regulatory regions, enhancers and transcription factor (TF) binding sites (TFBSs). Enhancers are cis-acting DNA sequences that can boost gene transcription and therefore play a critical role in regulating tissue-specific gene expression. They typically function independently of orientation and at varying distances from their target promoters [17]. TFBSs are another major class of non-coding regulatory regions. They are frequently found clustered in short sequences of 5–30 nucleotides within the promoters [18]. SNPs of these two types of regulatory regions can directly constitute an important part of regulation in the human genome through altered binding affinity for TFs. Since TFs recognize and bind specific DNA sequences and affect the expression of target genes, polymorphic variants located in TFBSs and enhancers could perturb transcription factor binding and eventually alter gene expression [19]. With this research, we sought to assess whether SNPs in these regulatory regions are germline cancer susceptibility gene variants in PDAC by using GWAS data.

## Material and methods

### Study populations

As the discovery population, genotyping data from PanScan I, PanScan II, PanScan III, and PanC4 were downloaded from the database of Genotypes and Phenotypes (dbGaP) website (study accession numbers: phs000206.v5.p3 and phs000648.v1.p1, project reference: #12644). All the individuals were genotyped using Illumina InfiniumHumanHap550v3 (PanScan I), Illumina InfiniumHuman610-Quad (PanScan II), OmniExpress arrays (PanScan III) or HumanOmniExpressExome-8v1 (PanC4) DNA Analysis Genotyping BeadChips. After merging the four genotype datasets (hereafter referred to as PanScan/PanC4), we performed imputation on the genotype data with the TOPMed imputation panel (version TOPMed-r2), followed by quality control steps. We excluded subjects with cryptic relatedness (PI_HAT > 0.2), gender mismatches, and variants with a minor allele frequency (MAF) < 0.01, completion rate and call rate < 98%, low-quality imputation score (INFO score < 0.7), evidence for violations of Hardy–Weinberg equilibrium (*p* < 1 × 10^−5^), leaving 7,509,345 variants genotyped on 14,266 individuals (7205 cases and 7061 controls) in the final dataset. PLINK 2.0 was used to perform principal component analysis on genotypes from all study populations, merged with genotypes of subjects from phase 3 of the 1000 Genomes Project. Individuals who did not cluster with the 1000 Genomes subjects of European descent in the principal component analysis (*N* = 439) were excluded from further analysis.

In order to narrow the list of variants, the summary statistics of a meta-analysis based on three East Asian studies [the Japan Pancreatic Cancer Research (JaPAN) consortium GWAS, the National Cancer Center (NCC) GWAS, and the BioBank Japan (BBJ) GWAS] comprising 2,039 pancreatic cancer patients and 32,592 controls in the Japanese population was used [20]. Genotyping on these individuals was performed using Illumina HumanCoreExome (JaPAN), Illumina HumanHap550/Illumina Human610-Quad (NCC) or Illumina HumanOmniExpressExome/Illumina HumanOmniExpress (BBJ) Genotyping BeadChips. Imputation was performed on each dataset with the 1000G phase3 v5 reference panel. A total of 7,914,378 variants remained after the post-imputation quality control, excluding variants with a MAF < 0.01 and low-quality imputation score (INFO score < 0.5).

A total of 7182 individuals (3392 PDAC cases and 3790 controls) from the PANcreatic Disease ReseArch (PANDoRA) consortium were genotyped to validate the previously selected variants. PANDoRA was previously described in detail [21]. It is a multicentric consortium consisting of 11 European countries (Greece, Italy, Germany, the Netherlands, Denmark, the Czech Republic, Hungary, Poland, Ukraine, Lithuania, and the UK), whose samples and data have been collected at the German Cancer Research Center (DKFZ, Heidelberg, Germany), where the DNA bank and the central database were established. PDAC cases were defined as individuals with an established diagnosis of PDAC. Controls were patients from the general population without any pancreatic disease at recruitment, individuals hospitalized for reasons other than cancer, or blood donors. Data were collected on sex, age, and country of origin for each case and control. Controls were recruited in the same geographical regions as the cases. Controls from the Netherlands and Germany were obtained respectively from the ‘European Prospective Investigation into Cancer and Nutrition’ (EPIC) [22] and the ‘Epidemiological investigations on chances of preventing, recognizing early and optimally treating chronic diseases in an elderly population’ (ESTHER) [23]. For this study, only PANDoRA subjects with self-declared European ancestry were included.

### SNP selection

In order to improve our chances of finding associations with PDAC risk, first of all we limited our selection to SNPs showing association at *p* < 10^−4^ in the PanScan/PanC4 dataset and not in LD among them [*r*^2^ > 0.6, checked with LDlink (https://ldlink.nci.nih.gov)], resulting into 2575 SNPs. The SNPs in TFBSs were retrieved from the SNP2TFBS database [24], containing annotations for 200 transcription factors with SNPs predicted to alter their affinity for binding. The effects of SNPs in the whole genome on TF binding were estimated using position weight matrices (PWM), which model the specificity of TF binding. The database calculates a score based on the difference between the PWM match scores of both alleles for each SNP-TF binding. The TFBS SNP list was generated by downloading all predicted variant-TF interactions from the SNP2TFBS database (*N* = 2,281,137, involving 1,900,881 unique SNPs).

To obtain enhancer SNPs, we used a defined list of enhancer regions from published research [25], which used the activity-by-contact (ABC) model to predict which enhancers regulate which genes in 131 human cell types and tissues. First, we extracted the genomic positions of enhancers and their target genes reported for the normal pancreatic tissue and the PANC-1 pancreatic cancer cell line. We thus obtained 55,967 enhancer regions. As the next step, we mapped SNPs on the enhancer regions via UCSC genome browser tools [26] to create a list of SNPs situated in enhancers consisting of 1,190,420 SNPs.

We checked the associations of polymorphisms in TFBS and enhancers with PDAC risk in the discovery dataset (PanScan/PanC4). In the following step, we performed a meta-analysis between TFBS SNPs and enhancer SNPs present in the results of both PanScan/PanC4 and East Asian GWAS summary statistics, using the “meta” and “metafor” R packages. We then excluded known pancreatic cancer risk loci and the SNPs in LD with them (*r*^2^ > 0.6). To select SNPs for the replication phase, we applied the following inclusion criteria: *p* < 10^−4^ in the meta-analysis and a significant association in both PanScan/PanC4 and the East Asian GWAS summary statistics (*p* < 0.05). In addition, we selected SNP rs17358295, because it showed the most significant association in the East Asian GWAS summary statistics (*p* = 4.6 × 10^−3^), despite having only a modestly significant association in the meta-analysis (*p* = 2.7 × 10^−2^). We finally picked the top significant SNPs in TFBSs and enhancers for genotyping analysis on the PANDoRA population. Summary information on the SNPs in TFBSs and enhancers selected for replication is included in Additional file 3: Data S3.

### Sample preparation and genotyping

The sample preparation and genotyping process were conducted at a single laboratory at German Cancer Research Center in Heidelberg, Germany. DNA extraction from the whole blood of both cases and controls within the PANDoRA consortium was carried out using a Qiagen-manufactured kit (Hilden, Germany). To ensure uniformity, the order of DNA samples from case and control subjects was randomized on plates, guaranteeing an equal representation of cases and controls in each batch. Genotyping was conducted via allele-specific PCR-based TaqMan technology (ThermoFisher, Applied Biosystems, Waltham, MA) by ordering TaqMan SNP Genotyping Assays for the selected seven SNPs. The PCR protocol was performed with TaqMan Genotyping Master Mix in 384-well plates following the manufacturer's recommendations. The PCR plates were read on a ViiA7 real-time instrument (Applied Biosystems), and genotypes were determined using the ViiA7 RUO Software, version 1.2.2 (Applied Biosystems).

### Statistical analysis

In the discovery phase, a logistic regression analysis was carried out by computing odds ratio (OR), 95% confidence intervals (95% CI), and *p* values to test the association between the SNPs and PDAC risk. The analysis was performed on 14,266 individuals (PanScan/PanC4) and was adjusted for sex, age, and the top ten principal components to avoid confounding due to population stratification. A meta-analysis was performed between the East Asian GWAS summary statistics and the discovery population. The top seven SNPs were analyzed in PANDoRA using logistic regression, adjusting for age, sex and country of origin (PANDoRA lacks GWAS data, therefore principal component data are not available). Deviation from Hardy–Weinberg equilibrium was tested for the variants genotyped in PANDoRA using the control subjects. Finally, a meta-analysis was conducted between the results of the three populations with a total of 56,079 individuals. Meta-analysis models were chosen depending on the heterogeneity (fixed-effect: *I*^2^ < 50%, random-effect: *I*^2^ ≥ 50%). In order to take into account the number of independent tests, LD, with a threshold of *r*^2^ > 0.6, was used to discard variants representing the same association. The Bonferroni-corrected threshold for statistical significance was 0.05/2575 = 1.94 × 10^−5^.

### Functional annotation

Several databases were utilized to link the variants with the best associations to potential functional explanations. To identify the possible effect of the SNPs on gene expression (eQTL/sQTL analysis), we used the data available in the Genotype-Tissue Expression (GTEx) project (https://www.gtexportal.org). We used the Ensembl Variant Effect Predictor (VEP) (https://www.ensembl.org/info/docs/tools/vep/index.html), HaploReg (https://pubs.broadinstitute.org/mammals/haploreg/haploreg.php), RegulomedB (https://www.regulomedb.org), Expression Atlas (https://www.ebi.ac.uk/gxa/home), The Human Protein Atlas (https://www.proteinatlas.org), TNMplot (https://tnmplot.com/analysis/) to check for regulatory potentials (for example, changes in transcription factor affinity, chromatin state regulation, changes in the expression). By using the Ensemble website (https://www.ensembl.org/), we analyzed the regions near the significant SNPs to look for regulatory regions.

## Results

In this study, we used the SNP2TFBS database and the published data [25] on genome-wide enhancer maps. These two datasets were used to establish two comprehensive lists of SNPs with a potential regulatory role. We checked their possible associations with PDAC risk with a two-phase approach, a discovery phase consisting of data on 7205 cases and 7061 controls from a GWAS conducted on PDAC risk (PanScan/PanC4) and 2039 cases and 32,592 controls from an East Asian GWAS, and a replication phase comprising 3392 PDAC patients and 3790 controls from the PANDoRA consortium. Therefore, the final sample size used in this study was 12,636 PDAC cases and 43,443 controls, as shown in Table 1.

**Table 1:** Characteristics of the study populations

	PanScan/PanC4	East Asian GWAS	PANDoRA
Total	14,266	34,631	7182
Cases	7205	2039	3392
Controls	7061	32,592	3790
Male%	54.80%	57.00%	54.20%
Median age (25–75% CI)	65.5 (55–75)	65.0	62.2 (53–71)
Number of variants*	7,509,345	7,914,378	7

*The number of variants remaining after quality control of GWAS data. For PANDoRA, the number of variants genotyped in the replication phase

**Fig. 1:**
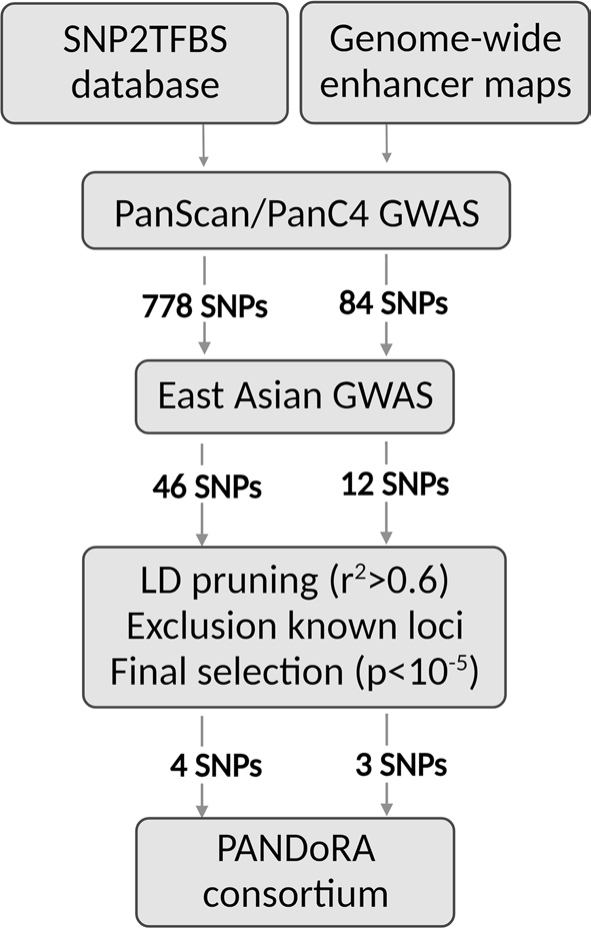
Summary of the variant selection workflow

**Fig. 2:**
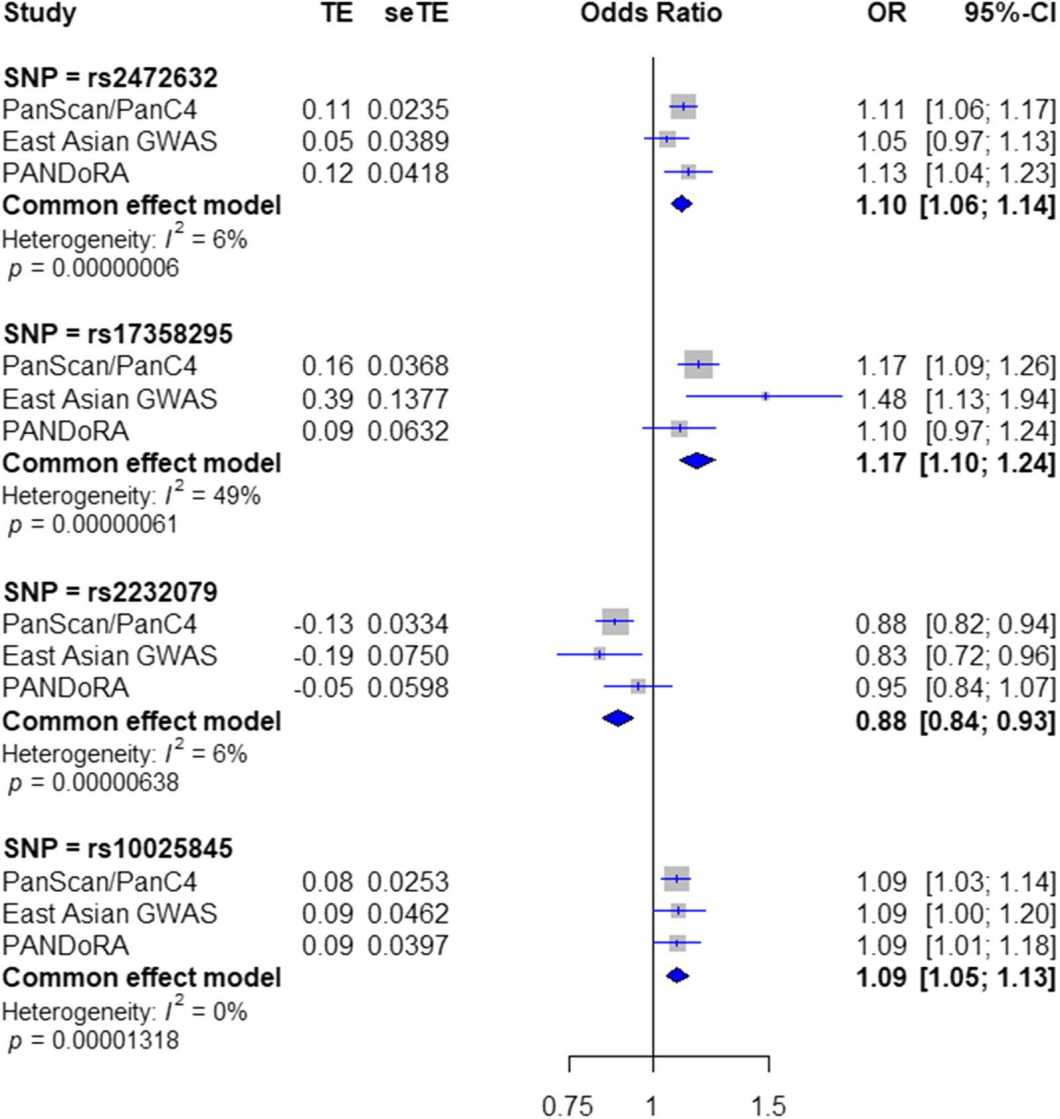
Summary of overall meta-analysis results for the four study-wise significant variants

The TFBS and enhancer SNP lists were first intersected with 2575 independent SNPs associated with PDAC risk with *p* < 10^−4^ from the PanScan/PanC4 dataset, which resulted in 778 TFBS SNPs and 84 enhancer SNPs. In the following step, we performed a meta-analysis between 673 TFBS SNPs and 12 enhancer SNPs which are present in PanScan/PanC4 and East Asian GWAS summary statistics. This resulted in 46 SNPs in TFBS and 12 in enhancers showing association with PDAC risk with *p* < 0.05. By considering a combination of low association *p* values and in-silico functional after exclusions of the known PDAC risk loci and SNPs in high linkage disequilibrium (LD; *r*^2^ > 0.6) (see materials and methods for details), we finally picked the top 4 significant SNPs in TFBSs (rs10025845, rs11241697, rs11032793, rs2232079) and the top 3 significant SNPs in enhancers (rs2472632, rs17358295, rs11624002) for genotyping analysis in PANDoRA. A scheme of SNP selection is shown in Fig. 1. The results of the overall meta-analysis for four study-wise significant SNPs are shown in Table 2 and Fig. 2. The associations between these seven SNPs and the risk of PDAC for all three populations are shown in Additional file 1: Data S1.

**Table 2:** Associations of the selected SNPs with PDAC risk

rsID	Locus	EA	NEA	EUR_freq	PanScan/PanC4		East Asian GWAS		PANDoRA		Overall meta-analysis	
					*p* value	OR [95%-CI]	*p* value	OR [95%-CI]	*p* value	OR [95%-CI]	*p* value	OR [95%-CI]
rs10025845	4p14	A	G	0.41	3.76 × 10^−4^	1.08 [1.03; 1.13]	3.43 × 10^−2^	1.09 [1.00; 1.18]	2.00 × 10^−2^	1.08 [1.01; 1.16]	**1.32 × 10**^−**5**^	1.08 [1.05; 1.12]
rs11241697	5q23.2	C	T	0.41	2.46 × 10^−4^	0.91 [0.87; 0.96]	4.68 × 10^−3^	0.89 [0.89; 0.96]	1.54 × 10^−1^	1.08 [0.96; 1.22]	3.67 × 10^−1^	0.95 [0.83; 1.06]
rs2472632	11p14.1	A	C	0.36	4.35 × 10^−6^	1.11 [1.06; 1.16]	2.01 × 10^−1^	1.05 [0.97; 1.13]	1.00 × 10^−3^	1.13 [1.05; 1.21]	**5.52 × 10**^**−8**^	1.10 [1.06; 1.13]
rs11032793	11p13	C	T	0.1	4.27 × 10^−5^	1.17 [1.08; 1.26]	3.77 × 10^−2^	1.37 [1.01; 1.85]	5.23 × 10^−1^	0.97 [0.91; 1.04]	2.69 × 10^−1^	1.10 [0.93; 1.27]
rs17358295	11p13	G	T	0.11	1.64 × 10^−5^	1.17 [1.09; 1.25]	4.60 × 10^−3^	1.47 [1.13; 1.93]	1.10 × 10^−1^	1.09 [0.97; 1.22]	**6.10 × 10**^**−7**^	1.16 [1.10; 1.22]
rs11624002	14q32.2	T	C	0.16	8.20 × 10^−5^	1.13 [1.06; 1.20]	1.84 × 10^−1^	1.07 [0.96; 1.19]	4.94 × 10^−1^	1.03 [0.94; 1.13]	1.04 × 10^−4^	1.09 [1.04; 1.14]
rs2232079	20p12.3	T	C	0.11	5.31 × 10^−4^	0.87 [0.81; 0.94]	3.94 × 10^−2^	0.83 [0.69; 0.99]	4.00 × 10^−1^	0.94 [0.83; 1.07]	**6.38 × 10**^**−6**^	0.88 [0.83; 0.93]

Values in bold are study-wise significant (*p* < 1.94 × 10^−5^)

*NEA* non-effect allele, *EA* effect allele, *EUR_freq* effect/minor allele frequency in European populations of the 1000 Genomes project

**Fig. 3:**
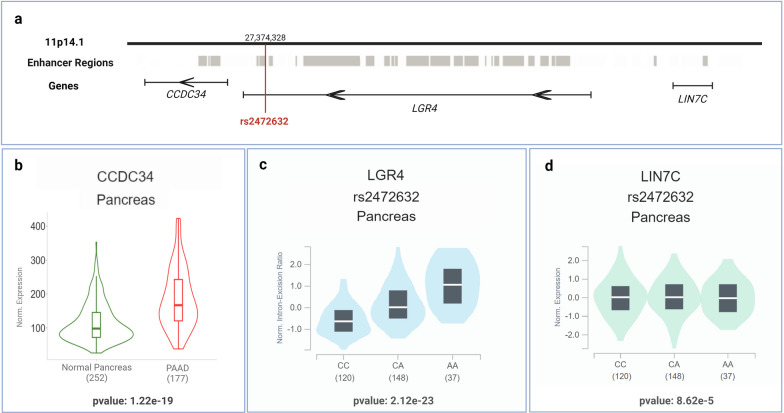
**a** Genomic location of the SNP rs2472632. **b** *CCDC34* gene expression in pancreas tissue from normal vs Pancreatic Adenocarcinoma patients in TNMplot database. **c** Violin plot of *LGR4*—rs2472632 sQTL analysis results in GTEx database. **d** Violin plot of *LIN7C*-rs2472632 eQTL analysis in GTEx database

rs2472632(A) was observed to be associated with increased PDAC risk at a nearly genome-wide significance level after the meta-analysis of all three populations (*p* = 5.52 × 10^−8^) with the same effect size direction in all populations. This enhancer region variant was predicted to affect expression of the coiled-coil domain containing 34 (*CCDC34*) gene (Fig. 3a), in normal pancreas tissue (ABC score = 0.016; computation of ABC scores is explained in the original paper [25]). In Pancreatic Adenocarcinoma (PAAD) tumor tissues, the *CCDC34* gene was significantly overexpressed with *p* = 1.22 × 10^−19^ compared to normal pancreas tissues (Fig. 3b) according to the TNMplot database, which has RNA-seq data of tumor and healthy tissues from TCGA and GTEx repositories.

Additionally, rs2472632(A) is located in the intron region of the leucine-rich repeat-containing G protein-coupled receptor 4 (*LGR4*) gene, the expression of which was 3.1 times higher in PDAC tissues than in normal pancreas tissues (false discovery rate-adjusted* p* = 1.92 × 10^−11^) in the Expression Atlas database. The splicing quantitative trait loci (sQTL) analysis in GTEx showed that the rs2472632(A) was associated with an alternative splicing mechanism of *LGR4* mRNA (*p* = 2.12 × 10^−23^) (Fig. 3c), and eQTL analysis in GTEx suggests that the rs2472632(A) was associated with higher expression of lin-7 homolog C (*LIN7C*) (*p* = 8.62 × 10^−5^) in the pancreatic tissue (Fig. 3d).

Furthermore, rs2472632(A) is associated with decreasing protein levels of defensin, alpha 5 (*DEFA5*), also known as human alpha defensin 5 (*HD5*), in the GWAS Catalog with *p* = 1.0 × 10^−25^ (Study accession: GCST90247261).

The enhancer variant rs17358295(G) with *p* = 6.1 × 10^−7^ and the TFBS variant rs2232079(T) with *p* = 6.38 × 10^−6^ were observed to be significantly associated with PDAC risk in the overall meta-analysis. While rs17358295(G) had an increased risk, the TFBS variant had a decreasing risk for PDAC in all three populations. rs17358295 maps to an enhancer that targets 20 genes in normal pancreas tissue, with ABC scores ranging between 0.015 and 0.130 (see Additional file 2: Data S2).

The TFBS variant, rs10025845(A), was associated (*p* < 0.05) in all three populations and became statistically significant at the study-wide level after overall meta-analysis (*p* = 1.35 × 10^−5^). While this variant’s minor allele (G) creates a binding site for the transcription factor *Yin Yang 1* (*YY1*)*,* the effect allele (A) is predicted by SNP2TFBS not to bind any TF. Additionally, this variant overlaps with a lincRNA coding gene, *LINC01258*.

The enhancer variant rs11624002(T) showed an association with PDAC risk in the overall meta-analysis, which however was not significant when considering a Bonferroni-corrected threshold. This variant is located within an enhancer near a protein-coding gene, Delta 4-Desaturase, Sphingolipid 2 (*DEGS2*), with ABC score = 0.018 (Additional file 3: Data S3).

Finally, two SNPs, rs11241697(C) and rs11032793(C), predicted to create/abrogate binding patterns for TFs, did not show statistically significant associations in the overall meta-analysis, including all cases and controls. Moreover, the meta-analysis showed high heterogeneity for them (*I*^2^ > 75%).

## Discussion

The majority of SNPs found to be associated with disease risk lie outside of protein-coding regions [27], which makes interpreting GWAS results challenging. This remains true even after fine mapping around the associated loci [28]. Most disease-associated variants affect gene expression by altering functional DNA elements. New tools are available for predicting the functional characteristics of non-coding variants. In this study, we leveraged recently developed advanced functional annotation to perform a comprehensive association analysis between non-coding variants and the susceptibility to PDAC.

The overall meta-analysis showed a variant, rs2472632(A), associated with PDAC risk close to genome-wide significance. The enhancer where rs2472632 is located is predicted to target the *CCDC34* gene, an oncogene that has been reported to be up-regulated in bladder cancer [29], cervical cancer [30], colorectal cancer [31], and PDAC [32]. Qi et al. and the TNMplot database, both showed by using the TCGA and GTEx datasets that *CCDC34* mRNA expression levels were significantly increased in PAAD compared with normal pancreatic tissues and were associated with significantly poor overall survival. This finding suggests that the A allele of the enhancer variant might increase the expression of the *CCDC34* oncogene by creating a stronger binding affinity for transcription factors in the locus. However, in the literature there are not yet functional studies available to elucidate the direct effect of the increased expression of this gene on cancer development mechanisms in PDAC cells.

Moreover, rs2472632 is located in an intronic region of *LGR4*, a gene that functions as an activator of the Wnt/β-catenin signaling pathway [33]. Although this pathway plays an important role during development, its abnormal activation has been reported as one of the predisposing factors in many cancer types, such as melanoma [34], multiple myeloma [35], ovarian cancer [36], thyroid carcinoma [37], etc. In addition, the Wnt/β-catenin signaling pathway promotes apoptosis resistance, which contributes to pancreatic cancer pathogenesis [38]. As a member of the signaling pathway, *LGR4* is best known for regulating the cells’ ability to respond to Wnt ligands and is widely expressed in the pancreatic tissue [39]. According to the sQTL analysis results, rs2472632 seems to be located at a splicing site of an alternative exon of *LGR4*. This finding suggests that the variant could contribute to PDAC risk through alternative RNA splicing. In addition, eQTL analysis showed that rs2472632 is associated with *LIN7C* expression, a membrane trafficking protein linked to metastasis development in some cancers [40]. Furthermore, deletions on 11p14.1, the chromosomal region where the enhancer variant is located, have been associated with attention deficit hyperactivity disorder (ADHD), developmental delay, and obesity [41].

Additionally, the rs2472632(A) variant has been associated with a reduction in HD5 peptide levels. HD5, a member of the defensin protein family, is a crucial antimicrobial peptide with powerful activity against various pathogens due to its ability to create pores in their membranes and enter their cytosol [42]. Notably, Paneth cells, located at the base of small intestinal crypts, release HD5 in response to stimuli like bacteria and cholinergic signals [43]. Also, we know that HD5 participates in the regulation of acute and chronic inflammatory processes [44]. This emphasizes the role of HD5 in reducing tissue inflammation, including pancreatic tissue. Indeed, in one study researchers demonstrated the presence of HD5 protein in PDAC tissues using immunohistochemistry [45]. In another study, researchers damaged the pancreatic duct in rats chemically and they found increased level of alfa-defensin-5 protein as a response [46]. The hypothesis posits that individuals carrying the A allele of the rs2472632 variant might exhibit lower levels of expressed HD5 protein. This potential decrease in HD5 expression could lead to reduced protection against both acute and chronic pancreatitis, conditions that have been linked to an increased risk of developing PDAC. Furthermore, in a healthy Japanese population (35–81 years old) HD5 concentration in fecal samples was significantly lower in the elderly group (age > 70 years old) than the middle-aged group (age ≤ 70 years old) [47]. This suggests that reduced levels of HD5 could be associated with an elevated risk of diseases in the elderly population. This also supports our hypothesis to make the point that having low HD5 levels could increase the risk of developing PDAC.

Thus, although the exact mechanism by which rs2472632 is associated with PDAC risk is not clear, various lines of evidence suggest that the locus and variations in this locus deserve more attention to be investigated functionally.

Our study pointed out two further loci significantly associated with PDAC risk, rs10025845(A) and rs2232079(T). While the minor allele (A) of rs10025845 is not predicted to result in any TF binding site, the G allele creates a binding site for the YY1 TF, which has also been shown to play a tumor suppressor role in PDAC [48]. This finding suggests that, as a result of the A allele, the ability to suppress tumors by YY1 might be reduced. The other variant we identified in our study, rs2232079, is located at the binding site of TF *Paired Box 5* (*PAX5*) and the promoter region of *Fermitin family member 1* (*FERMT1*), which encodes Kindlin-1. The Kindlin-1 protein is overexpressed in pancreatic cancer cell lines but only expressed at a low level in normal pancreatic epithelial cells and fibroblasts [49].

According to the enhancer map data, rs17358295(G) maps to an enhancer with 20 different target genes by activity-binding-contact with a range of ABC scores. The highest ABC score (0.130) for this variant was with the ETS homologous factor gene (*EHF*), whose roles in cancer remain largely unknown. Some studies showed that the overexpression of EHF protein plays a role in metastasis, proliferation, and shorter survival rates in cancer patients [50–52].

Several GWAS published in recent years have found that most disorders are associated with only a few common SNPs, and even when considered as a whole, their associated SNPs explain only a small percentage of the risk. Going beyond the analysis of GWAS primary results, performing secondary analyses on existing GWAS data has proven to help further our understanding of genetic risk factors which makes it possible to achieve better risk stratification.

The strengths of our study are its large sample size with multiple ethnicities and its comprehensive evaluation of the two major functional classes of polymorphisms, which led to our finding of a germline variant with a nearly genome-wide significance level for PDAC risk. On the other hand, one of the limitations of this study was the data we used for retrieving TFBS SNPs is only based on in silico predictions, and likewise for enhancers SNPs, our prediction of possible polymorphism function is based only on position. Another limitation is the lack of experimental validation of our findings. In the future, this limitation could be addressed by in vitro experiments with CRISPR-Cas9-edited cell lines. These experiments aim to assess SNP impacts on gene expression and pathways, revealing insights into differential binding to transcription factors and resulting expression. Integrating functional genomics, transcriptomics, and proteomics provides a comprehensive understanding. Optimal setting to perform these experiments would be creating healthy pancreatic ductal cells from induced pluripotent stem cell (iPSC) cultures [53].

## Conclusions

With this study, we discovered several novel promising germline genetic risk loci for genetic susceptibility to PDAC, which are candidates for experimental functional validation.

### Supplementary Information

**Additional file 1. **Data S1 Detailed information of overall meta-analysis result in all-three populations.

**Additional file 2.** Data S2 ABC scores and targeted genes of the selected enhancer SNPs.

**Additional file 3.** Data S3 Detailed information of selected variants from discovery phase.

## Data Availability

The PanScan and PanC4 genotyping data are publicly available from the database of Genotypes and Phenotypes (dbGaP, study accession numbers phs000206.v5.p3 and phs000648.v1.p1). The summary statistics of the East Asian GWAS are publicly available at http://www.aichi-med-u.ac.jp/JaPAN/current_initiatives-e.html. The PANDoRA primary data for this work will be made available to researchers who submit a reasonable request to the corresponding author, conditional to approval by the PANDoRA Steering Committee and Ethics Commission of the Medical Faculty of the University of Heidelberg, Germany. Data will be stripped from all information allowing the identification of study participants.

## Abbreviations

ABC: Activity-by-contact
ADHD: Attention deficit hyperactivity disorder
BBJ: BioBank Japan
*CCDC34*: *Coiled-coil domain containing 34*
dbGaP: Database of genotypes and phenotypes
*DEGS2*: *Delta 4-desaturase, sphingolipid 2*
*EHF*: *ET* *S* *homologous factor gene*
EPIC: European Prospective Investigation into Cancer and Nutrition’
eQTLs: Expression quantitative trait loci
ESTHER: Epidemiological investigations on chances of preventing, recognizing early and optimally treating chronic diseases in an elderly population
*FERMT1*: *Fermitin family member 1*
GTEx: The genotype-tissue expression
GWAS: Genome-wide association studies
*HD5*: *Human alpha defensin 5*
JaPAN: Japan Pancreatic Cancer Research
*LGR4*: *Leucine-rich repeat-containing G protein-coupled receptor 4*
*LIN7C*: *Lin-7 homolog C*
lncRNAs: Long noncoding RNAs
NCC: National Cancer Center
MAF: Minor allele frequency
OR: Odds ratio
PAAD: Pancreatic adenocarcinoma
PanC4: Pancreatic Cancer Case–Control Consortium
PANDoRA: The PANcreatic Disease ReseArch Consortium
*PAX5*: *Paired box 5*
PDAC: Pancreatic ductal adenocarcinoma
PWM: Position weight matrices
SNP: Single-nucleotide polymorphisms
sQTL: Splicing quantitative trait loci
TCGA: The cancer genome atlas
TFBS: Transcription factor binding site
UCSC: University of California, Santa Cruz
*YY1*: *Yin Yang 1*
